# Interventional Photothermal Therapy Enhanced Brachytherapy: A New Strategy to Fight Deep Pancreatic Cancer

**DOI:** 10.1002/advs.201801507

**Published:** 2019-01-15

**Authors:** Fengrong Zhang, Xianlin Han, Yanyan Hu, Shunhao Wang, Shuang Liu, Xueting Pan, Hongyu Wang, Junjie Ma, Weiwei Wang, Shanshan Li, Qingyuan Wu, Heyun Shen, Xiaoling Yu, Qipeng Yuan, Huiyu Liu

**Affiliations:** ^1^ Beijing Advanced Innovation Center for Soft Matter Science and Engineering State Key Laboratory of Organic–Inorganic Composites Bionanomaterials & Translational Engineering Laboratory Beijing Key Laboratory of Bioprocess Beijing Laboratory of Biomedical Materials Beijing University of Chemical Technology Beijing 100029 China; ^2^ Department of General Surgery Peking Union Medical College Hospital Beijing 100730 China; ^3^ Medical Department First Affiliated Hospital of PLA General Hospital Beijing 100048 China; ^4^ State Key Laboratory of Environmental Chemistry and Ecotoxicology Research Center for Eco‐Environmental Sciences Chinese Academy of Sciences Beijing 100085 China; ^5^ Department of Interventional Ultrasound General Hospital of People's Liberation Army Beijing 100853 China

**Keywords:** biodegradable, brachytherapy, honeycomb‐like gold, interventional photothermal therapy, pancreatic cancer

## Abstract

Photothermal–radiotherapy (PT–RT) is an effective strategy for relieving hypoxia‐related radiotherapy resistance and inducing tumor‐specific cell apoptosis/necrosis. Nevertheless, limited tissue penetration of near‐infrared (NIR) laser and the serious side effects of high‐dose radiation severely hinder its applications for deep tumors. An interventional photothermal–brachytherapy (IPT–BT) technology is proposed here for the internal site‐specific treatment of deep tumors. This technology utilizes a kind of biodegradable honeycomb‐like gold nanoparticles (HGNs) acting as both internal photothermal agents and radiosensitizers. A high tumor inhibition rate of 96.6% is achieved in SW1990 orthotopic pancreatic tumor‐bearing mice by HGNs‐mediated IPT–BT synergistic therapy. Interestingly, this approach effectively causes double‐stranded DNA damage and improves the oxygen supply and the penetration of nanoparticles inside the tumor. Therefore, it is believed that this strategy may open up a new avenue for PT–RT synergistic therapy of deep malignant tumors and has a significant impact on the future clinical translation.

## 1 Introduction

Pancreatic cancer (PC) remains one of the most lethal malignancies worldwide with limited diagnosis, poor prognosis, and rapid metastasis.^1^ In clinical practice, external beam radiotherapy (RT) is a standard therapeutic modality for PC with advantages of less pain, curative effect, and easy combination with other therapies.^2^ Nevertheless, the high dose of radiation may exceed the surrounding normal tissues tolerance and lead to nonselective collateral damage.^3^ Meanwhile, the inadequate oxygen supply inside the deep tumor may cause hypoxia‐associated RT resistance,^4^ resulting in a less therapeutic potential.

Thermoradiotherapy is an effective strategy to enhance tumoral blood flow and microvascular permeability, which may reduce the proportion of hypoxic cells at an early tumor, inducing tumor site vascular disruption/collapse and necrosis.^5^ Previous reports demonstrated gold nanoparticles (GNs) with an obvious localized surface plasmon resonance (LSPR) peak in near‐infrared (NIR) region and strong photoelectric absorbance capacities of X‐rays could be applied for tumor photothermal–radiotherapy (PT–RT).^6^ However, the limited tissue penetration depth of NIR laser light severely hinder its applications for deep tumors, due to the difficulty to deliver enough laser energy into malignant tissues deeper than 10 cm.^7^

To overcome these problems, we hypothesized that interventional photothermal therapy (IPTT) combined with brachytherapy (BT) (IPT–BT) could improve the therapeutic efficacy. BT is a standard localized internal radioisotope therapy realized by in situ implanting radioactive seed with minimal invasion.^8^ IPTT technology has recently been developed in our group for PC treatment with precise heating and reduced tissue injuries.^9^ As far as we know, a technique which could combine IPTT and BT, whose energy source both come from the device inside the body, has not been attempted in deep cancer treatment.

**Scheme 1:**
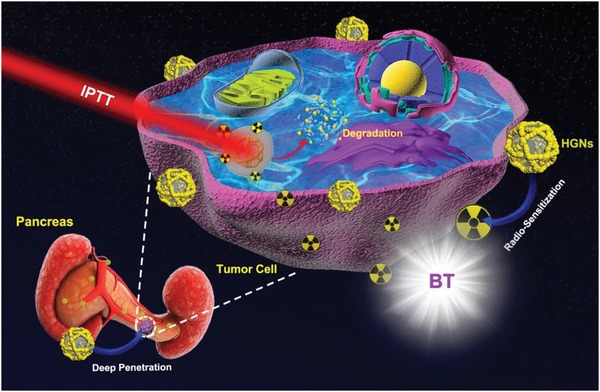
Schematic illustration of biodegradable HGNs‐mediated IPT–BT synergistic therapy for PC.

Herein, we first developed an IPTT‐enhanced iodine‐125 (^125^I) BT synergistic therapy technology to treat PC. In detail, we synthesized biodegradable honeycomb‐like gold nanoparticles (HGNs) composed of liposomes and gold, endowing the nanostructure with NIR radiation absorbance and X‐ray attenuation ability. Compared with reported PT–RT, HGNs‐mediated IPT–BT synergistic strategy provides the specific advantages as follows: 1) Implanted low‐dose‐rate ^125^I seed reduced damage to normal tissues as well as strengthened the energy accumulation of radiation at the tumor site.^8^ 2) IPTT could access deep abdominal cavity for localized treatment, reducing the laser power attenuation caused by percutaneous irradiation of traditional photothermal therapy (PTT).^9^ 3) The honeycomb‐like porous gold nanoparticles could be decomposed into less than 5 nm within 24 h in simulated lysosomal fluid (SLF), giving a chance for in vivo renal clearance of HGNs.^10^ The detail therapeutic principle based on HGNs is presented in **Scheme** **1**. The improved blood flow, induced by the tumor‐site photothermal effect, greatly enhanced intratumoral accumulation and penetration of HGNs. Meanwhile, hyperthermia induced by HGNs also increased oxygen supply by blood flow, which could reduce hypoxia‐associated RT resistance and promote HGNs sensitized DNA double‐strand breaks. Briefly, we successfully developed a biodegradable HGNs‐mediated IPT–BT synergistic therapy for PC, achieving 96.6% tumor inhibition rate in SW1990 orthotopic pancreatic tumor–bearing mice. We believe that this technology has a significant potential for patients with deep and unresectable tumors.

## 2 Results and Discussion

**Figure 1:**
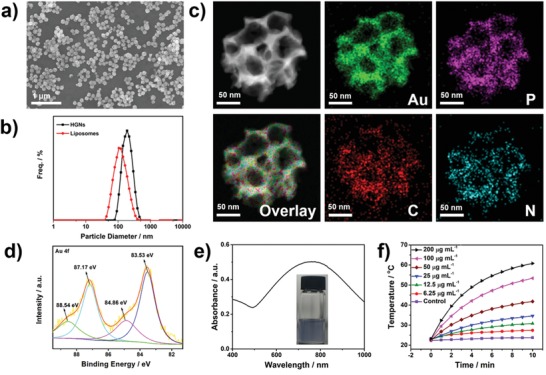
Characterization of HGNs. a) SEM image of HGNs. b) Size distribution of HGNs and liposomes. c) Elemental mapping for gold, phosphorus, carbon, and nitrogen. d) Au 4f XPS spectra and e) UV–vis–NIR absorption spectrum of HGNs. The inset is a solution color photograph of HGNs in deionized water. f) Temperature elevation of HGNs at different concentrations (0, 6.25, 12.5, 25, 50, 100, and 200 µg mL^−1^) under laser irradiation (808 nm, 1 W cm^−2^, 10 min).

Biodegradable HGNs were fabricated via an in situ reduction strategy according to the earlier report.^11^ And the tunable honeycomb‐like morphology was further obtained by changing the mass ratio of Au precursor and reductant (Figure S1, Supporting Information). Scanning electron microscopy (SEM) image revealed that the used HGNs were monodispersed with uniform sizes of about 150 nm in diameter, which are suitable for passive tumor accumulation according to the enhanced permeability and retention (EPR) effect (**Figure** **1**a).^12^ The average hydrodynamic diameters of HGNs and liposomes were 175 and 112 nm measured by dynamic light scattering (DLS), respectively (Figure 1b). The spectrum of energy‐dispersive X‐ray spectroscopy (EDS) of HGNs showed peaks assigned to gold and carbon elements, indicating the existence of the gold and liposome (Figure S2, Supporting Information). The element mapping identified that the gold, phosphorus, carbon, and nitrogen elements homogeneously distributed in the bulk of HGNs (Figure 1c). In addition, the X‐ray photoelectron spectroscopy (XPS) for Au 4f chemical states were performed to observe the binding energy changes of metallic gold from HAuCl_4_. Two remarkable double peaks of Au 4f_5/2_ and Au 4f_7/2_ at 87.17 and 83.53 eV were assigned to Au^0^.^13^ The other two weak peaks at 88.54 and 84.86 eV could be attributed to the Au 4f_5/2_ and Au 4f_7/2_ of Au^+^ state (Figure 1d; Figure S3, Supporting Information).^14^ These results revealed that the formation of HGNs could be attributed to the stepwise in situ reduction route of gold, from Au^3+^ to Au^+^, then to Au^0^.

NIR absorption property of photothermal agents is critical for NIR‐mediated PTT. As shown in Figure 1e, the ultraviolet‐visible‐infrared (UV–vis–NIR) absorption spectrum of HNGs revealed that it has strong NIR absorption peak. In Figure 1f and Figure S4a in the Supporting Information, the temperature of HGNs aqueous dispersions increased in time‐ and concentration‐dependent manners under NIR laser radiation. Compared with deionized water, the temperature of HGNs at 200 µg mL^−1^ solution boosted to 60 °C, which is much higher than the tolerable temperature of tumor cells.^15^ The photothermal performance of HGNs did not show obvious deterioration after one month of storage (Figure S4b, Supporting Information). Besides, the photothermal conversion efficiency of HGNs was calculated to be ≈26.9% (Figure S5a,b, Supporting Information), higher than previously reported gold nanoparticles (e.g., gold nanovesicle (18%)^16^ and gold nanoshells (17.5%)^17^). The extinction coefficients of HGNs was calculated to be 7.02 L g^−1^ cm^−1^ via Lambert–Beer law (Figure S5c, Supporting Information). The resulting live/dead staining also validated HGNs could act as a good mediator for PTT (Figure S6, Supporting Information).

The degradation feature of HGNs is favorable for their excretion from the body through the renal glomerular basement membrane after therapy.^16^, ^18^ That has been found to be associated with inherent physicochemical properties of HGNs as well as pH in the tumor microenvironment. To better understand the degradation process, HGNs were treated in different simulated physiological environments. In the simulated body fluid (SBF) with an approximately neutral pH, the morphology or dispersity of HGNs did not have any distinct change in 12 h. But in an SLF with pH 4.5, significant change of HGNs was observed at the same time point. The porous morphology had almost been destroyed. It is reported that the degradation of gold–liposome nanocomplex could be attributed to the hydrolysis of β‐ester bond in glycerol‐linked phospholipids, which was the main pathway for lipid breaking down and recycling in the physiological conditions.^11^ The smaller gold particles with a diameter of about 5 nm in SLF at 24 h indicated the decomposition of HGNs, whereas some lipid fragments could be discerned due to the agglomeration of hydrolyzed acyl tails. These data proved the biodegradation properties of HGNs in the biological simulation system, and which was accelerated by the SLF (Figure S7, Supporting Information).

**Figure 2:**
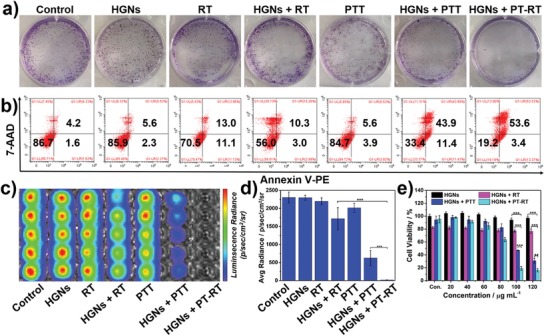
In vitro HGNs‐mediated PT–RT synergistic therapy. a) The colony of SW1990 cells treated with HGNs (100 µg mL^−1^) combined with laser irradiation (808 nm, 1 W cm^−2^, 3 min) and X‐ray radiation (6 Gy). b) Corresponding flow cytometry–based apoptosis analysis of SW1990 cells with the same treatments. c) Representative bioluminescence images based on cell death in various groups. d) Quantitative analysis of bioluminescence (*n* = 5). e) Relative viabilities of SW1990 cells with different treatments. **P < 0.001, ***P < 0.0001.

To test the role of HGNs in the IPT–BT synergistic therapy, we conducted a clonogenic survival assay, a standard method for evaluating the radiosensitization efficiency of HGNs in vitro. It has been reported that RT could effectively induce DNA damage in mitochondria, which is highly correlated with the radiation resistance of cancer cells.^19^ So we incubate SW1990 cells and PANC‐1 cells with HGNs for 24 h upon NIR and X‐ray, to yield an elevated intracellular level of singlet oxygen (^1^O_2_) originated from mitochondria. Opposed to cells without HGNs, SW1990 cells and PANC‐1 cells treated with HGNs after X‐ray irradiation presented less viable cell colonies, confirming the radiosensitization effect of HGNs (**Figure** **2**a; Figure S8, Supporting Information). Furthermore, rare colony formation in HGNs‐mediated PT–RT group explained that HGNs have the potential both as photothermal agents and radiosensitizers. To confirm this result, those cells were analyzed by flow cytometry with the help of fluorescent intercalating agents, Annexin V‐phycoerythrin (Annexin V‐PE) and 7‐amino‐actinomycin D (7‐AAD). SW1990 cells and PANC‐1 cells in HGNs + PT–RT group showed notably enhanced late apoptosis ratio (53.6% and 67.2%, respectively) compared with other groups (Figure 2b; Figure S9, Supporting Information). This result was additionally supported by direct observation of the firefly luciferase 2 (fluc2) luminescence SW1990‐fluc2 cells (Figure 2c,d) because the fluc2 enzyme catalyzed its substrate D‐luciferin only in the presence of cellular adenosine triphosphate (ATP).^20^ The standard 3‐(4,5‐dimethylthiazol‐2‐yl)‐2,5‐diphenyltetrazolium bromide (MTT) assay is also performed for assessing the cellular cytotoxicity in different groups. After incubation with HGNs for 24 h, no apparent cytotoxicity was discerned even at the concentration up to 120 µg mL^−1^. By comparison, when exposed to NIR laser, the cell viabilities dramatically decreased with the increasing HGNs concentration and 120 µg mL^−1^ HGNs solution caused about 70% cells death under 808 nm NIR irradiation, illustrating the effective photothermal killing ability of HGNs. It was also found that 76.68% and 30.89% cells endured after the treatments of HGNs + RT (***P < 0.0001) and HGNs + PTT (**P < 0.001), while more than 80% of cells were dead when treated with HGNs + PT–RT, at 120 µg mL^−1^ of HGNs (Figure 2e). The calculated combination index (CI), a quantitative parameter of combination effects, was 0.65 based on the cell viability of HGNs + RT, HGNs + PTT, and HGNs + PT–RT groups, indicating synergistic effects of PT–RT.^21^ In addition, γ‐H2AX, a maker for DNA double‐strand break, was utilized to evaluate DNA damage caused by HGNs‐mediated PT–RT. Consistent with the above results, SW1990 cells treated with HGNs after X‐ray irradiation presented less viable DNA damage, while the highest expression level of γ‐H2AX was observed in HGNs + PT–RT group (Figure S10, Supporting Information). Our data evidenced that PT–RT synergistic therapy mediated by HGNs has great potential in killing PC cells compared to other single therapeutic modalities.

**Figure 3:**
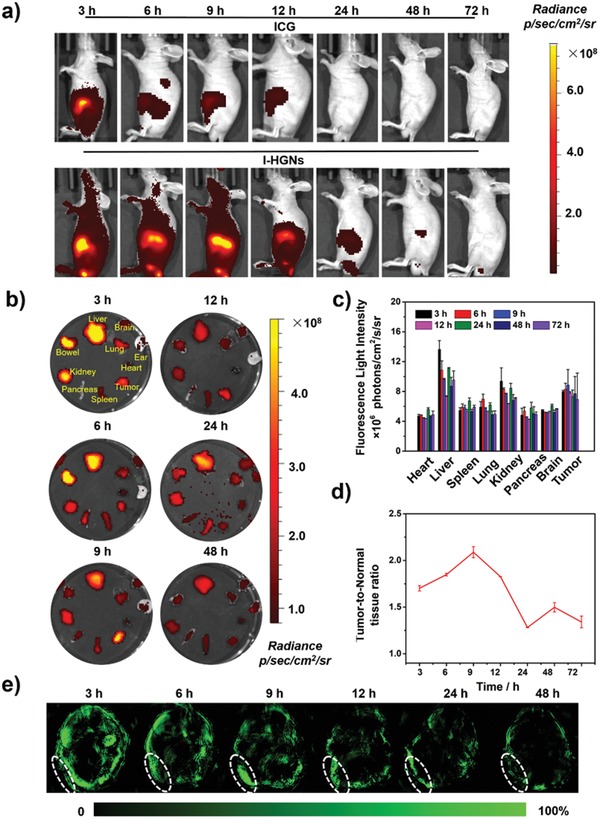
Biodistribution of HGNs in SW1990 pancreatic tumor–bearing mice in vivo. a) The biodistribution of ICG or I‐HGNs after tail vein injection. b) In vitro fluorescence images of tumors and major organs collected from tumor‐bearing mice with I‐HGNs injection at different time points. c) Quantitative analysis of fluorescence in major organs and tumor tissues. d) Tumor‐to‐normal tissue ratio of fluorescence intensity at different time points after tail vein injection of HGNs. e) PA images of mice after injection of HGNs (1 mg mL^−1^, 100 µL) via the tail vein at different time points.

To study in vivo biodistribution of HGNs, we used SW1990 pancreatic tumor–bearing mice injected by HGNs conjugated with fluorescent dye indocyanine green (I‐HGNs) to analyze their behaviors in vivo. As shown in **Figure** **3**a, I‐HGNs reached up to their peak accumulation in tumor lesions after 9 h post injection, which confirmed a longer in vivo circulation time than that of free indocyanine green (ICG). Meanwhile, tumor and other major organs (heart, liver, spleen, lung, kidney, pancreas, brain, and bowel) were excised at different time points (3, 6, 9, 12, 24, and 48 h) for fluorescence quantification analysis. The strongest signal at 9 h post injection was observed, indicating the efficient passive tumor targeting ability of I‐HGNs (Figure 3b,c). Besides, the measurement of fluorescence signals and the inductively coupled plasma mass spectrometry (ICP‐MS) of pancreatic tumors and main organs further confirmed this result, presenting the optimal time window for subsequent treatments (Figure 3d; Figure S11, Supporting Information). Intense distribution of gold element in kidney and urine implied that HGNs might be corrupted into small gold particles with low pH and eliminated through a renal removal pathway, which is consistent with previous studies.^18^, ^22^ We also identified the photoacoustic (PA) signals mediated by HGNs in SW1990 pancreatic tumor–bearing mice model. Due to the effective HGNs accumulation in tumor, PA signals in the tumor region notably improved at 9 h post injection, in accordance with the fluorescence and ICP‐MS results (Figure 3e; Figure S12, Supporting Information). These data not only suggest that a large amount of HGNs homogenously accumulated inside the tumor after systemic administration, but also indicate that HGNs are good PA imaging agents based on the acoustic waves generation induced by the NIR absorbance performance of HGNs, allowing increased spatial resolution in optical imaging during the treatment process.

**Figure 4:**
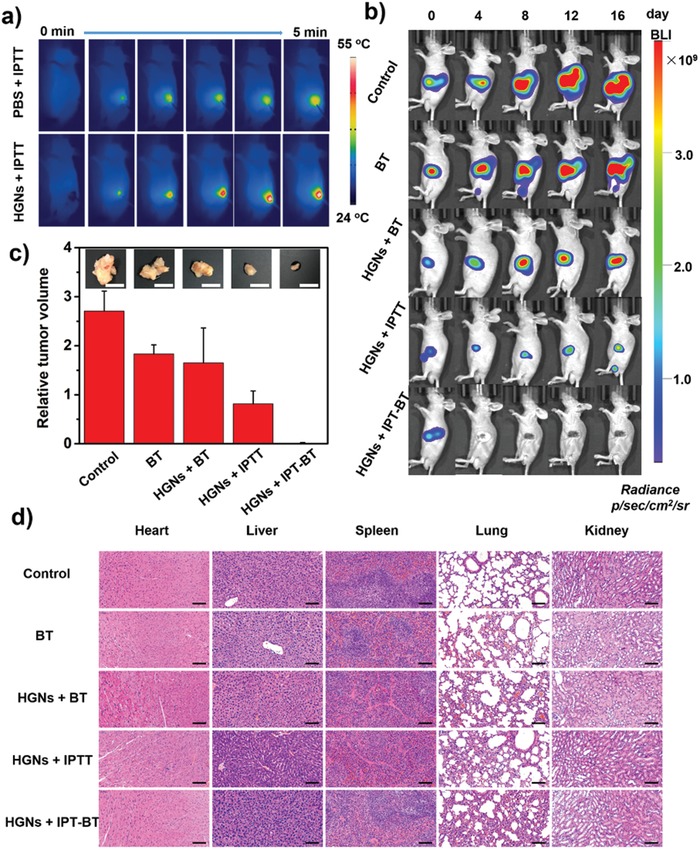
In vivo IPT–BT synergistic therapy of SW1990 pancreatic tumor–bearing mice. a) Thermographs of mice administrated with PBS (100 µL) or HGNs (250 µg mL^−1^, 100 µL) exposed to NIR irradiation (2.0 W cm^−2^, 5 min). b) Bioluminescence images of tumor‐bearing mice post various treatments. c) Relative tumor volume in the group of control, BT, HGNs + BT, HGNs + IPTT, and HGNs + IPT–BT groups on day 16 post treatment. Insets: corresponding digital pictures of tumor post treatments. Scale bars are 1 cm. d) H&E stained images of major organs after therapy. Scale bars are 100 µm.

Motivated by the effective synergistic effects of HGNs‐mediated PT–RT in vitro, the in vivo synergistic therapeutic effects were further investigated in SW1990 pancreatic tumor–bearing mice under the guidance of multimodal imaging. Firstly, we evaluated the changes of temperature at the tumor site under 808 nm laser radiation by an infrared thermal imager (**Figure** **4**a). Compared to phosphate buffered saline (PBS) injection group (≈43 °C), the tumor temperature in the treatment group increased quickly and reached up to ≈55 °C within 5 min (Figure S13, Supporting Information). The relative higher tumor temperature in the treatment group than those in the control group could effectively kill cancer cells. For studying the synergistic therapeutic effects in vivo, the mice were randomly divided into five groups (*n* = 8 per group): 1) Control group (PBS injection); 2) BT group (one ^125^I seed implantation); 3) HGNs + BT group (HGNs injection and one ^125^I seed implantation); 4) HGNs + IPTT group (HGNs injection and laser radiation); and 5) HGNs + IPT–BT group (HGNs injection, laser radiation, and one ^125^I seed implantation). In the treatments involving BT, one ^125^I seed was implanted into the tumor sites under the guidance of ultrasound imaging (Figure S14, Supporting Information). The bioluminescence imaging (BLI) was used to monitor the tumor growth at 4‐day intervals continuously. A slight decrease of BLI signal was observed in HGNs + BT group at 16th day post administration when compared with BT alone, which demonstrated that the remarkable radiation effect with the aid of nanoparticles. Nevertheless, a strong fluorescence signal could still be observed at the tumor site. This may be contributed to the hypoxia‐associated BT resistance, which decreased the killing efficiency for cancer cells by BT. Notably, the HGNs + IPTT group and HGNs + IPT–BT group showed significant advantages in tumor destruction, but HGNs + IPT–BT group is more efficient in preventing locoregional recurrence (Figure 4b), indicating that the effective therapeutic effects based on HGNs‐mediated IPT–BT synergistic therapy. To accurately analyze the synergistic treatment efficiency, the mice were sacrificed after 16 days post administration, and the tumor volume was detected. As shown in Figure 4c, HGNs + IPT–BT group showed 96.6% tumor inhibition rate, consisting with BLI data. The pathological examination by hematoxylin and eosin (H&E) of tissue slices indicated that there was no significant apoptosis/necrosis in major organs in all groups (Figure 4d). Moreover, the hemolysis rate after incubating with HGNs at different concentrations are much less than the limited upper value of hemolysis index (5%), indicating that the obtained HGNs had excellent biocompatibility (Figure S15, Supporting Information). Subsequently, to better understand the potential toxicity caused by HGNs, blood biochemistry and hematology analysis of healthy BALB/c mice were also performed for 16 days after intravenous (i.v.) injection. The main parameters of liver function, kidney function, and hematology analysis showed no obvious change, compared with the untreated group (Figure S16, Supporting Information). All these evidence strongly pointed to the fact that IPT–BT synergistic therapy based on HGNs was helpful for eliminating the deep tumors and improving hypoxia‐associated BT resistance with no side effects to normal tissues, which might play an important role in PC treatment.

**Figure 5:**
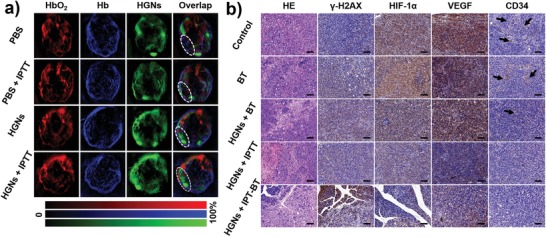
In vivo mechanism of IPT–BT synergistic therapy. a) PA images of tumor distribution pre‐ and posttreatment with PBS (100 µL) or HGNs (1 mg mL^−1^, 100 µL), and with NIR irradiation for 5 min (2.0 W cm^−2^) or without. The tumor regions were highlighted by white dashed circles. b) Representative H&E stained and immunohistochemical images of tumor slices collected from mice in various groups. The black arrows in the inset of the CD34 group indicate remained CD34 on blood vessels. Scale bars are 100 µm.

To further investigate the mechanism of IPT–BT synergistic therapy, the PA images of mice were used to detect the change of tumor microenvironments such as blood flow, oxygen supply, and HGNs contents in different tumor depth. The signal intensities from oxyhemoglobin (HbO_2_) obviously increased under the laser irradiation in HGNs + IPTT group (**Figure** **5**a), which resulted from hyperthermia‐mediated enhanced oxygenation status by increasing the blood supply in tumor tissue, but there was nearly no change in the PBS‐treated group. As shown in Figure 5b, the yellowish brown color of tumor tissue turned pale after treated with HGNs + IPT–BT, suggesting that HIF‐1α, an intrinsic marker of hypoxia, was downregulated, and tumor tolerance decreased for RT due to hypoxia environment.^23^ In hypoxic tissue, HIF‐1α can not only promote vascular endothelial growth factor (VEGF) transcription but also increase the stability of VEGF,^24^ thereby upregulating VEGF expression, which was also consistent with HIF‐1α expression. Moreover, CD34 is a positive marker of total endothelial cells in new microvasculature, and promotes tumor invasion as well as metastasis.^25^ Notably, the expression of CD34 was upregulated in the control group, whereas large depletion of vessel structure was seen in HGNs + IPTT group and HGNs + IPT–BT group. In addition, the PA signal was detected in the whole tumor tissue in HGNs + IPTT group, indicating that more HGNs surrounding the blood vessels infiltrated into the tumor center after laser irradiation. To investigate whether HGNs could induce DNA double‐strand damage by IPT–BT synergistic effects in vivo, the expression levels of γ‐H2AX was also observed via immunohistochemical photomicrographs.^26^ As shown in Figure 5b, the yellow–brown area was observed to be largest in the HGNs + IPT–BT group, which reflected the damage of double‐stranded DNA by IPTT and ionizing radiation. In addition, quantitative analysis of the immunostaining was shown in Figure S17 in the Supporting Information. Therefore, HGNs‐mediated IPT–BT synergistic therapy could effectively cause damage of double‐stranded DNA, increase the supply of blood flow and oxygen, and enhance penetration of HGNs at the tumor sites.

## 3 Conclusion

In conclusion, we present a new technology of the IPT–BT synergistic therapy with photoactivated and radiosensitized HGNs for deep tumor treatment. The site‐specific technology is activated at deep abdominal cavity tumor via accumulated HGNs as an endogenous sensitizer of interventional NIR and the internal radioactive source. HGNs exhibited good degradability and biocompatibility, strong NIR longitudinal LSPR absorption, excellent X‐ray attenuation ability, with no adverse side effects. HGNs‐mediated IPT–BT efficiently increased utilization efficiency in tumor site of the laser depending on the interventional system, enhanced BT by attenuating X‐ray, and reduced side effects to the normal tissue via localized treatment. Although we focused on PC in this study, we believe that this strategy is also suitable for other deep lethal solid tumors and may open up the possibilities for the clinical translation of IPTT.

## 4 Experimental Section

*Materials*: 1,2‐dioleoyl‐sn‐glycero‐3‐phosphoethanolamine (DOPE), 1,2‐dipalmitoyl‐sn‐glycero‐3‐phosphocholine (DPPC), and 1,2‐dipalmitoyl‐sn‐glycero‐3‐phosphoethanolamine‐N‐[methoxy(polyethylene glycol)‐2000] (DSPE‐PEG_2000_) were obtained from Avanti Lipids Polar Company (Tullamarine, Australia). Cholesterol (CHOL), N‐hydroxysuccinimide (NHS), and potassium carbonate (K_2_CO_3_) were obtained from Sinopharm Chemical Reagent Co., Ltd (Beijing, China). 1‐ethyl‐3(3‐dimethylaminopropyl) carbodiimide hydrochloride (EDC), hydroxylamine hydrochloride, MTT, crystal violet, and ICG were purchased from Sigma (St. Louis, MO, USA). Tetrachloroauric acid trihydrate (HAuCl_4_·3H_2_O) was obtained from Shenyang Jinke Reagent Factory (Shenyang, China). All chemicals were used as received without further treatment.

*Characterization*: DLS was measured by Zetasizer Nano‐ZS (Malvern Instruments, UK). An HT‐7700 electron microscope was used to characterize the morphology of HGNs. Element mapping and EDS experiments were performed by APOLLO XLT2 transmission electron microscope. An S‐4700 SEM was used to characterize the size and morphology of HGNs, and XPS (Axis HSi, Kratos Ltd., UK) was used to analyze surface chemistry of HGNs. UV–vis absorption spectrum was obtained by 2600 UV‐Visible Spectrophotometer (SHIMADZU, Japan).

*Preparation of HGNs*: HGNs were prepared by an in situ reduction method according to the earlier report.^11^ Firstly, the mixtures containing DPPC (20 mg), DOPE (1 mg), DSPE‐PEG_2000_ (1 mg), and CHOL (6 mg) were dissolved in a chloroform solution (5 mL). The solution was then dried in a rotary evaporator under vacuum at 40 °C; then a thin lipid film was obtained. Later, the obtained film was hydrated with high‐intensity sonication at 60 °C after adding PBS. Liposomes with uniform size were prepared. And liposomes were further coated with gold in the PBS solution at room temperature. HAuCl_4_·3H_2_O (0.1 mg) and K_2_CO_3_ (2.5 mg) were added into 5 mL of liposome solution, stirred at room temperature for 4 h. Next, 0.3 mg of NH_2_OH·HCl was added into the above solution and stirred for another 10 min. The final product was collected by centrifugation and washed five times with PBS solution to remove the excess HAuCl_4_·3H_2_O, liposomes, and reducing agents.

*In Vitro Synergistic Therapy*: The clonogenic survival assay was first used in the evaluation of the therapeutic effects of HGNs on PC cells. SW1990 cells and PANC‐1 cells were seeded in 6‐well plates with a density of 1 × 10^5^ per well, and then seven groups were set (Control, HGNs, RT, HGNs + RT, PTT, HGNS + PTT, HGNs + PT–RT). After 24 h, cells were incubated with HGNs (100 µg mL^−1^) for 24 h. Subsequently, the cells were irradiated with 808 nm laser (1 W cm^−2^, 3 min) and irradiated by X‐ray (6 Gy). Then the cells were incubated for another 7 days, followed by being washed twice with PBS, fixed in 4% formaldehyde, and stained with crystal violet. Apoptosis assay was performed with a density of 2 × 10^5^ per well, and other treatments were taken under the same conditions as above. Then the cells were labeled with Annexin V‐PE/7‐AAD (Multisciences Biotech Co,. Ltd, Hangzhou, China), and evaluated via flow cytometry. Bioluminescence imagines were also carried out to analyze the synergistic effects of HGNs‐mediated PT–RT in vitro. SW1990 cells with a density of 1 × 10^4^ were plated per well in 96‐well plates, and other conditions were consistent with clonogenic assay. Later the cells were stained with 100 µL D‐luciferin substrate (50 µg mL^−1^, AnaSpec, Inc., Fremont, CA, USA) for 8 min and imaged by IVIS Spectrum Imaging System (PerkinElmer, Waltham, MA, USA). The light output was delivered by IVIS Lumina II imaging system.

*Cell Viability Assay*: SW1990 cells were plated with a density of 1 × 10^5^ per well in 96‐well plates and divided into four groups (HGNs, HGNs + RT, HGNS + PTT, HGNs + PT–RT). After 24 h, cells were incubated with HGNs for another 24 h, and then exposed to NIR laser (808 nm, 1 W cm^−2^, 3 min) and X‐ray (6 Gy) radiation. The MTT assay was conducted following the standard protocol.^27^

*Tumor Model*: BALB/c nude mice, male, aged 6–8 weeks, were obtained from Beijing Vital River Laboratory Animal Technology Co. Ltd and used in compliance with a local ethics committee (Permit Number: 2011‐0039). The pancreas of the BALB/c nude male mice was orthotopically injected with SW1990 cells (5 × 10^6^ cells, dilated in 100 µL PBS). The mice were ready for experiments when the xenografted pancreatic tumors reached 70 mm^3^.

*In Vivo PA Imaging*: PA signals of living mice were captured by real‐time MSOT (MSOT inVision 128, iThera medical, Germany). When the tumor reached 70 mm^3^, SW1990 pancreatic tumor–bearing mice injected with 100 µL of HGNs‐PBS solution (1 mg mL^−1^) were anesthetized with isoflurane, and PA signals of HbO_2_, hemoglobin (Hb), HGNs were recorded at different times.

*In Vivo Synergistic Therapy*: SW1990 pancreatic tumor–bearing mice were randomly divided into five groups (*n* = 8 per group): a) Control group (PBS injection), b) BT group (one ^125^I seed), c) HGNs +BT group (250 µg mL^−1^, 100 µL, one ^125^I seed), d) HGNs + IPTT (250 µg mL^−1^, 100 µL, 5 min, 2.0 W cm^−2^), e) HGNs + IPT–BT (250 µg mL^−1^, 100 µL, 5 min, 2.0 W cm^−2^, one ^125^I seed). The therapeutic radioisotopes ^125^I seed (29.6 MBq, 0.8 mCi) was implanted into tumor sites by a minimally invasive procedure. The IPTT device consisted of an NIR laser fiber (0.8 mm, Banglei Optoelectronic Technology Co., Ltd, Guangdong, China) and a custom hollow percutaneous transhepatic cholangiography needle (Zhuhai Hokai Biomedical Electronics Co., Ltd., China), which was penetrated into the deep abdominal pancreas. At the same time, the ultrasound was designed to check the position of the ^125^I seed. For evaluating synergistic therapeutic effects of IPT–BT, IVIS Spectrum Imaging System was utilized to monitor the tumor growth at 4‐day intervals continuously. All of the mice were sacrificed at 16 day after administration. Tumor volume was calculated via *ab* ^2^/2, where *a* and *b* represent the length and width of a tumor, respectively. The H&E of major organs (heart, liver, spleen, lung, and kidney) and tumor tissues were performed to assess the biocompatibility and antitumor efficiency of HGNs. All of the tissues were fixed in 4% neutral buffered paraformaldehyde, embedded routinely in paraffin, labeled with H&E. The H&E images were obtained with optical microscopy.

*Immunohistochemical Staining Analysis*: The expression levels of γ‐H2AX, HIF‐1α, VEGF, and CD34 were observed via immunohistochemical photomicrographs for exploring the mechanism of IPT–BT synergistic therapy. SW1990 tumor–bearing mice were divided into five groups previously. After various treatments, the tumors were isolated from mice and then fixed in neutral buffered paraformaldehyde, embedded routinely in paraffin. Next, the tumor sections were embedded in paraffin, the immunohistochemical analysis of γ‐H2AX, HIF‐1α, VEGF, and CD34 of tumors were performed as the protocol. In addition, the images were further analyzed with Image J software.

*Statistical Analysis*: All data were presented as the mean ± standard deviation or mean. Statistical analysis was conducted using *t*‐test. *P* values less than 0.01 were considered statistically significant.

## Conflict of Interest

The authors declare no conflict of interest.

## Supporting information

Supplementary
