# Supplementary material for: Interventional Photothermal Therapy Enhanced Brachytherapy: A New Strategy to Fight Deep Pancreatic Cancer
Source: Adv Sci (Weinh). 2019 Jan 15;6(5):1801507. doi: 10.1002/advs.201801507 (PMC6402393; doi:10.1002/advs.201801507)
Supplement: Supplementary file 1 — Supplementary [file ADVS-6-1801507-s001.pdf]

Copyright WILEY-VCH Verlag GmbH & Co. KGaA, 69469 Weinheim, Germany, 2019.

ADVANCED SCIENCE
Open Access

Supporting Information

for *Adv. Sci.,* DOI: 10.1002/advs.201801507

Interventional Photothermal Therapy Enhanced Brachytherapy: A New Strategy to Fight Deep Pancreatic Cancer

*Fengrong Zhang, Xianlin Han, Yanyan Hu, Shunhao Wang, Shuang Liu, Xueting Pan, Hongyu Wang, Junjie Ma, Weiwei Wang, Shanshan Li, Qingyuan Wu, Heyun Shen, Xiaoling Yu, Qipeng Yuan, and Huiyu Liu**

Copyright WILEY-VCH Verlag GmbH & Co. KGaA, 69469 Weinheim, Germany, 2016.

Supporting Information

Interventional Photothermal Therapy Enhanced Brachytherapy: A New Strategy to Fight Deep Pancreatic Cancer

*Fengrong Zhang, Xianlin Han, Yanyan Hu, Shunhao Wang, Shuang Liu, Xueting Pan, Hongyu Wang, Junjie Ma, Weiwei Wang, Shanshan Li, Qingyuan Wu, Heyun Shen, Xiaoling Yu, Qipeng Yuan, and Huiyu Liu**

Preparation of honeycomb-like gold nanoparticles conjugated with indocyanine green (I-HGNs). Firstly, 1 mg of honeycomb-like gold *nanoparticles* (HGNs) were mixed with 200 μL of thioglycolic acid (TGA; J&K Chemical Ltd., Beijing, China) in 3 mL of ultrapure water. After 4 h, the precipitates were washed three times with deionized water and collected after 10-min centrifugation at 10000 rpm. Then, 3 mL of deionized water was supplemented, and the pH of the suspension was adjusted to 6.0 by hydrochloric acid. Subsequently, N-hydroxysuccinimide (58 mM, 200 μL) and 1-ethyl-3(3-dimethylaminopropyl) carbodiimide hydrochloride (200 mM, 200 μL) were added and vibrated for 1 h at 30 °C, 2 mg of polyoxyethylene bis (amine) (NH_2-PEG-NH_2) was added into the mixture. After 3 h, the precipitates were collected and washed twice with phosphate buffered saline (PBS). Next, the precipitates were resuspended in 3 mL of ultrapure water and added 300 μg of indocyanine green (ICG). The mixture was then vibrated at 4 °C overnight, and I-HGNs were obtained by centrifugation again. To determine the conjugation efficiency of ICG, the final products were collected and calculated by the equation: conjugation efficiency of ICG (%) = (drug input – free drug in supernatant) /drug input × 100%. The final conjugation efficiency of ICG was 30.2%.

In vitro photothermal measurement. A series of HGNs aqueous dispersions (1 mL) with different concentrations were irradiated with an 808 nm laser (1 W cm^{-2}) for 10 min, and the

temperature change was recorded by an Infrared Thermal Imager (FLIR SC 620; FLIR system, Inc., Wilsonville, OR, USA) and thermocouple (TES 1315, TES Electrical Electronic Corp., Taiwan, China). As for the recycling heating profiles of HGNs, the aqueous dispersion (100 μg mL^{-1}, 1 mL) was irradiated with the 808 nm laser (1 W cm^{-2}) for four cycles, and each cycle consisted for 5 min followed by a 5 min cooling.

Calculation of the photothermal conversion efficiency. According to the expression of Roper,[1] the total energy balance of the whole system is:

$$\sum_i m_i C_{p,i} \frac{dT}{dt} = Q_{HGNs} + Q_{Dis} - Q_{out} \quad (1)$$

Where m and Cp mean the mass and heat capacity of water. In addition, T is the temperature of the solution. Q_{HGNs} is the photothermal energy inputted by HGNs. Q_{Dis} is the heat obtained by sample cell under 808 nm laser irradiation and Q_{out} is the heat transferred from the system.

$$Q_{HGNs} = I(1 - 10^{-A_{808}})\eta \quad (2)$$

Where I represent the density of laser power, A_{808} is the absorbance of HGNs dispersed in water at 808 nm and η is the photothermal conversion efficiency.

$$Q_{out} = hS\Delta T \quad (3)$$

Where h is the heat-transfer coefficient, S is the surface area of the container and ΔT is surrounding ambient temperature.

To obtain the value of hS, we further studied the cooling stage after the temperature reached a steady. When the laser turns off, $Q_{HGNs} + Q_{Dis} = 0$,

$$\sum_i m_i C_{p,i} \frac{dT}{dt} = -Q_{out} = -hS\Delta T \quad (4)$$

In order to acquire the hS, we introduced a dimensionless driving force temperature, θ to replace $\frac{\Delta T}{\Delta T_{max}}$

$$dt = -\frac{\sum_i m_i C_{p,i}}{hS} \frac{d\theta}{\theta} \quad (5)$$

And rearranging to obtain Equation 6:

$$t = \frac{\sum_i m_i C_{p,i}}{hS} \ln \theta \quad (6)$$

According to these Equations,

$$\eta = \frac{hS\Delta T_{max} - Q_{Dis}}{I(1-10^{-A_{808}})}$$

(7)

To evaluate the value of η, the temperature change of 1.0 mL HGNs aqueous dispersion (50 μg mL^{-1}) was recorded as a function of time irradiated with an 808 nm laser. The laser was turned off until the temperature reached a steady state. So we obtained the ΔT_{max} is 21.1 °C, Q_{Dis} is 77.8 mW, A_{808} is 0.562. In addition, the m is 0.3 g, and the C is 4.2 J g^{-1} $°C^{-1}$. Substituting these values into equation (7), the photothermal conversion efficiency of HGNs was calculated approximately to be 26.9%.

In vitro degradation. To carry out the in vitro degradation, HGNs (200 μg mL^{-1}) were added into simulated lysosomal fluid (SLF, 20 mL, pH = 4.5) and simulated body fluid (SBF, 20 mL, pH = 7.4), respectively, at 37 °C with a shaking rate (200 rpm). After different incubation time (6, 12, 24, 36, and 48 h), the samples were analyzed by transmission electron microscope (TEM).

Cell culture. The human pancreatic adenocarcinoma cell lines (SW1990 and PANC-1) were obtained from the Peking Union Medical College Hospital (Peking, China). Dulbecco's Modified Eagle Medium (DMEM), antibiotic-antimycotic solution, trypsin-EDTA solution, fetal bovine serum (FBS) and 1% penicillin/streptomycin were purchased from Corning (NY, USA). The cell lines maintained in DMEM containing 10% FBS and 1% penicillin/streptomycin at 37 °C.

Live/dead assay. SW1990 cells (1×10^5 per well) were seeded in 4-well plates for 24 h, then 100 μg mL^{-1} of HGNs dissolved in culture medium was added. After 24 h incubation, cells were irradiated with near-infrared (NIR) laser (808 nm, 1 W cm^{-2}) for 3 min, and further incubated for another 12 h. Finally, the cells were stained with tetraacetoxymethyl ester

(Calcine AM)/Propidium iodide (PI) (KeyGEN Biotech, Nanjing, China) following the protocol and observed by a Leica SP5 confocal laser scanning microscope (CLSM).

DNA double-strand damage evaluation. SW1990 cells (1×10^4 per well) were preseeded into confocal dishes for 24 h and then treated with HGNs (100 μg mL^{-1}). After 24 h incubation, those cells were subjected to irradiation of NIR laser (808 nm, 1 W cm^{-2}, 3 min) and X-ray (6 Gy) and further incubation for 12 h. Then, the culture medium was removed, and the cells were incubated with anti-γ-H2AX rabbit monoclonal antibody (KeyGEN Biotech, Nanjing, China) and then with Alexa Fluor 555-conjugated sheep anti-rabbit secondary antibody according to the protocol. In addition, cell nuclei were stained with Hoechst 33342 and observed by the CLSM.

Inductively-coupled plasma mass spectrometry analysis. Tumors and major organs were weighed, then dissolved in a solution containing 3:1 (v:v) hydrogen chloride and nitric acid, and the contents of gold in tumor and major organs were analyzed.

In vivo biodistribution. Tumor growth of the mice was monitored via IVIS Spectrum Imaging System (PerkinElmer, Waltham, MA, USA) after intraperitoneal injection of D-luciferin (15 mg mL^{-1}; AnaSpec, Inc., Fremont, CA, USA). SW1990 pancreatic tumor-bearing mice were intravenously (i.v.) injected by I-HGNs (100 μg mL^{-1}, 100 μL) to analyze the in vivo behaviors. A series of fluorescence images were collected at 3, 6, 9, 12, 24, 48, and 72 h post injection. In addition, quantification of the fluorescence intensity of tumor tissues and major organs (heart, liver, spleen, lung, kidney, pancreas, brain, and bowel) was carried out and calculated at different time points using the region of interest (ROI) functions of the IVIS Lumina II imaging system. To further analyze passive tumor targeting ability of HGNs, the tumor to normal tissue contrast ratios (T/N) was calculated using the ROI functions of the software at different time points.

Hemolysis evaluation. The blood from BALB/c nude mice was used to evaluate the hematotoxicity of HGNs in vitro. First, the red blood cells were separated by centrifugation at

2000 rpm for 10 min, and then washed with PBS 3 times. Subsequently, the red blood cells were diluted to 1/10 of their volume with PBS. Then different concentrations of HGNs: (a) 20 μg mL^{-1}, (b) 40 μg mL^{-1}, (c) 60 μg mL^{-1}, (d) 80 μg mL^{-1}, (e) 100 μg mL^{-1} were added into 1.2 mL of the cells solutions. The mixtures were kept still at 37 °C for 4 h, followed by centrifugation at 3000 rpm for 10 min. The supernatant was collected and measured absorbance at 570 nm. The hemolysis ratio was calculated as follows: dividing the difference in absorbance between the sample and the negative control by the difference in absorbance between positive and negative controls, and multiplying the resulting ratio by 100.

Blood biochemistry and hematology analysis. The blood samples were collected from healthy BALB/c nude mice at day 16 after i.v. injected with HGNs at doses from 25 to 100 mg kg^{-1}, and the samples from other healthy mice without any treatment were used as the control. All samples were tested to obtain the blood biochemistry and hematology data.

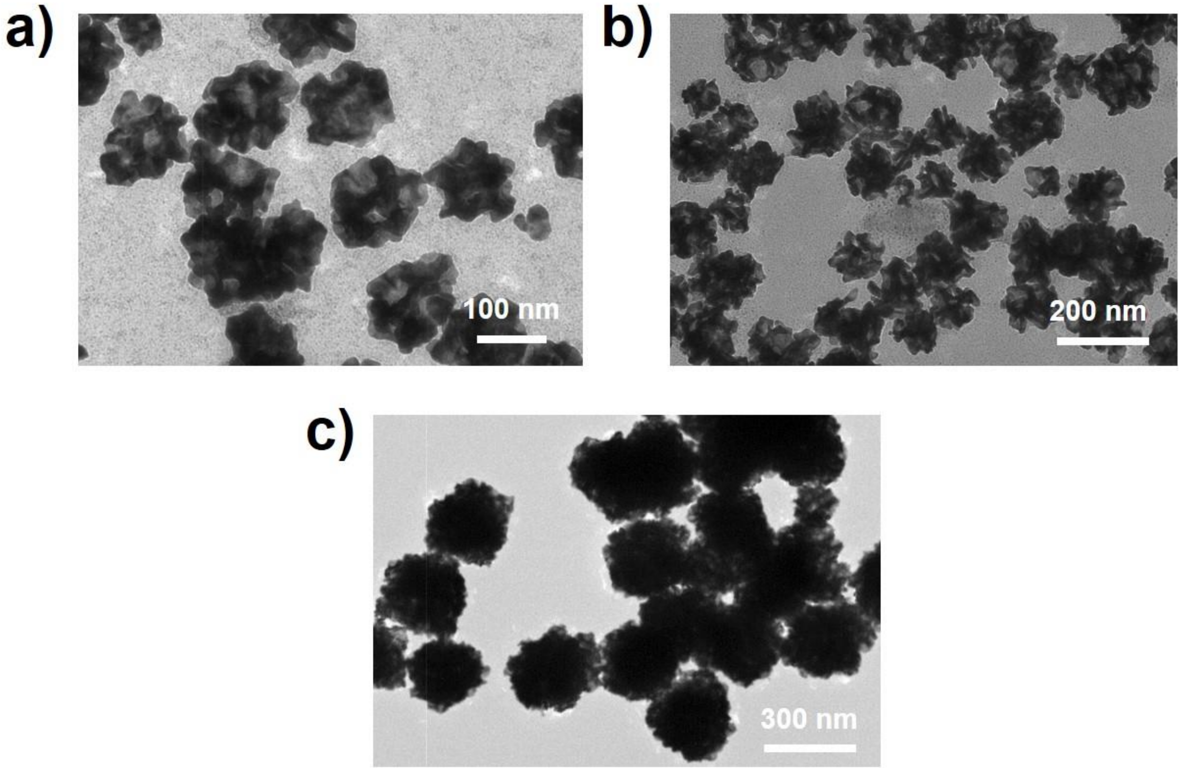

Figure S1. TEM images of HGNs with different volumes of hydroxylamine hydrochloride a) 5 μL, b) 10 μL, and c) 20 μL.

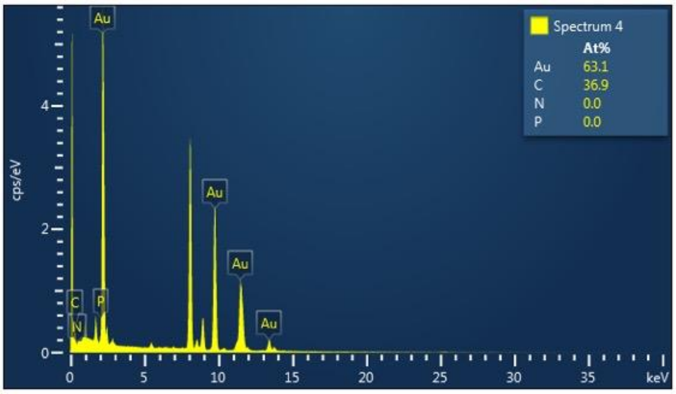

Figure S2. EDS spectrum of HGNs.

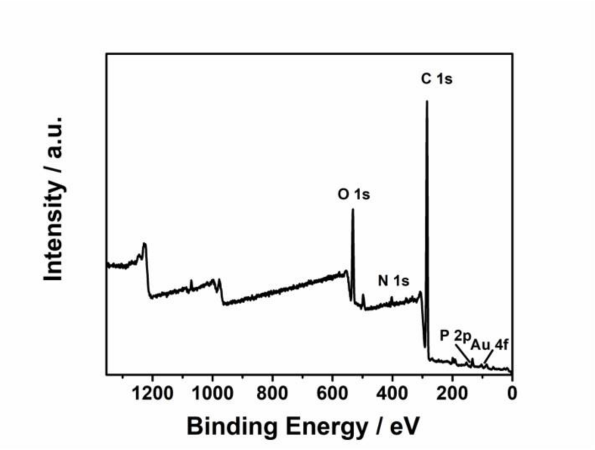

Figure S3. XPS spectrum of HGNs.

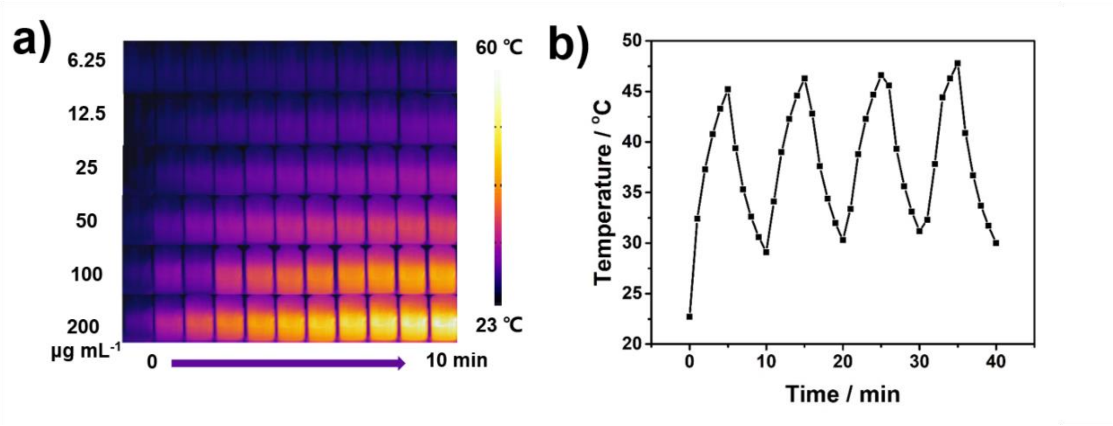

Figure S4. a) Infrared thermal images of HGNs at different concentrations (0, 6.25, 12.5, 25, 50, 100, and 200 μg mL^{-1}) under laser irradiation (808 nm, 1 W cm^{-2}, 10 min). b) Recycling heating profiles of HGNs (100 μg mL^{-1}) after one month of preparation using an 808 nm laser (1 W cm^{-2}).

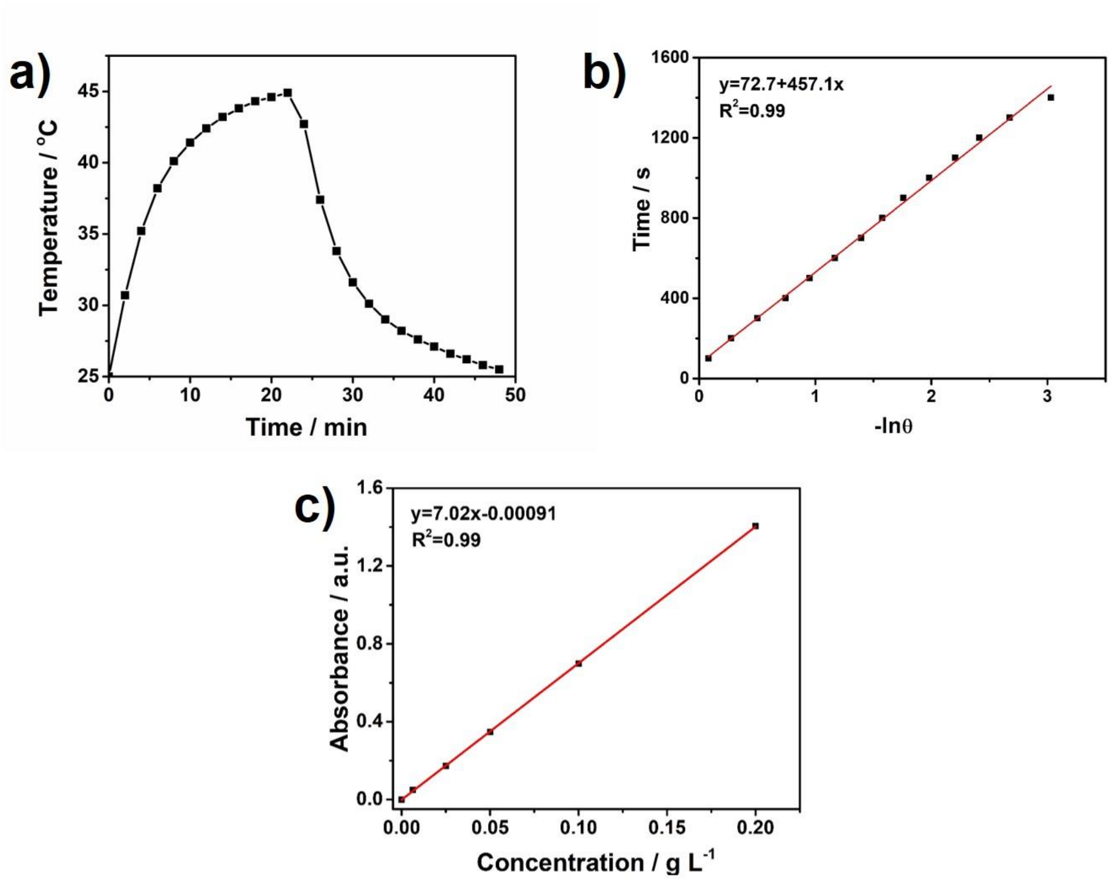

Figure S5. a) Photothermal response of HGNs (50 μg mL^{-1}) irradiated with an 808 nm laser. The laser was turned off until the temperature reached a steady state. b) Calculation of the time constant for heat transfer using a linear regression of the cooling profile. c) The UV-vis-NIR absorption and fitting curve of the absorption values at 808 nm of HGNs dispersions at different concentrations in water.

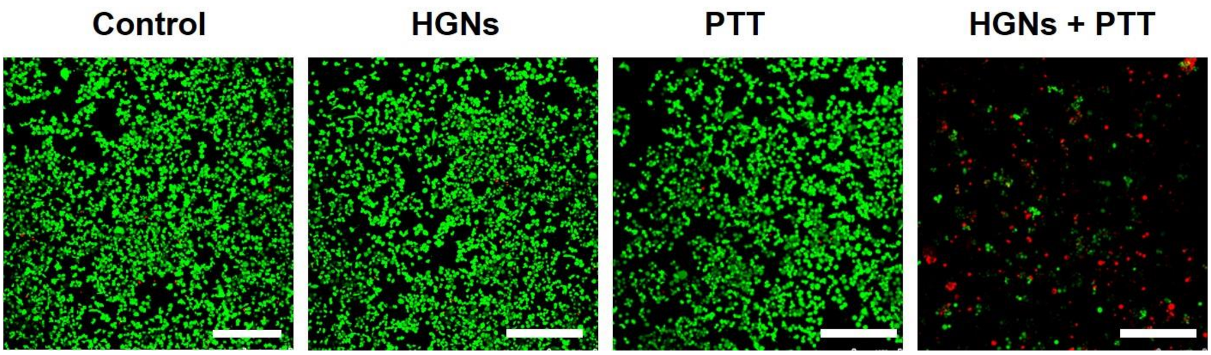

Figure S6. Live/dead staining of SW1990 cells incubated with HGNs (100 μg mL^{-1}) and treated with or without NIR laser irradiation (1 W cm^{-2}, 3 min). Green fluorescence shows live cells, and red fluorescence shows dead cells. Scale bars are 250 μm.

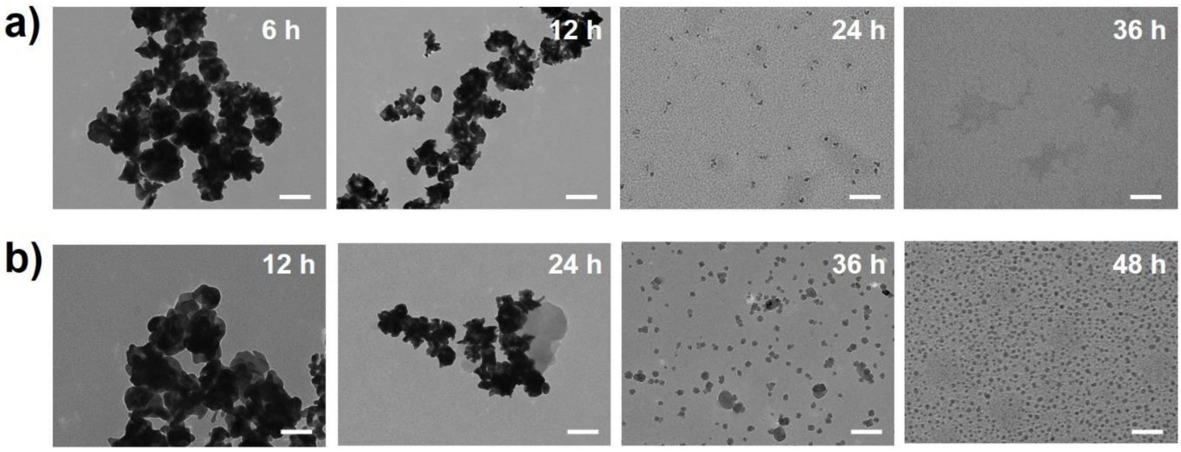

Figure S7. Degradation behaviors of HGNs in a) SLF and b) SBF observed by TEM. Scale bars are 100 nm.

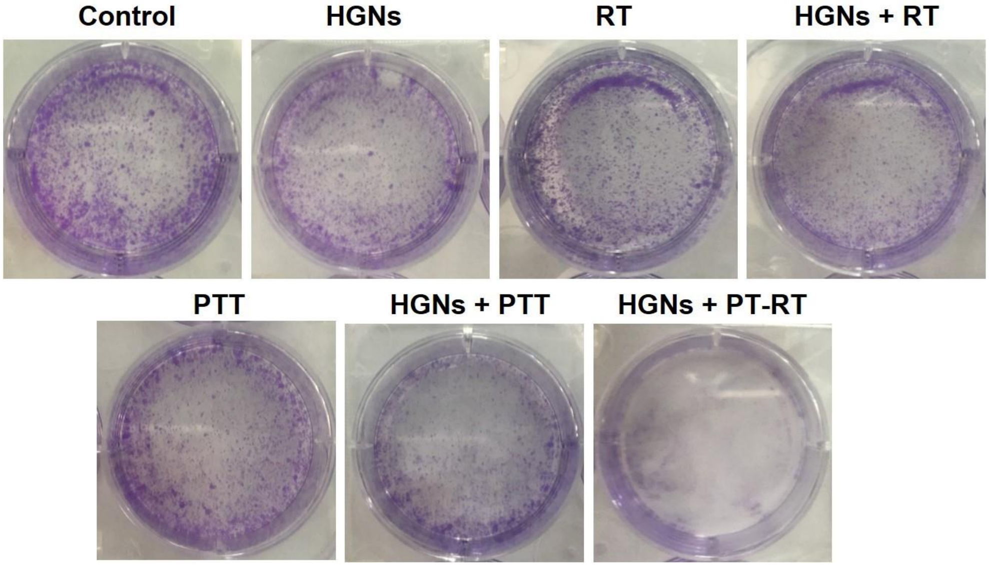

Figure S8. Colony of PANC-1 cells induced by HGNs (100 μg mL^{-1}) combined with laser irradiation (808 nm, 1 W cm^{-2}, 3 min) and X-ray radiation (6 Gy).

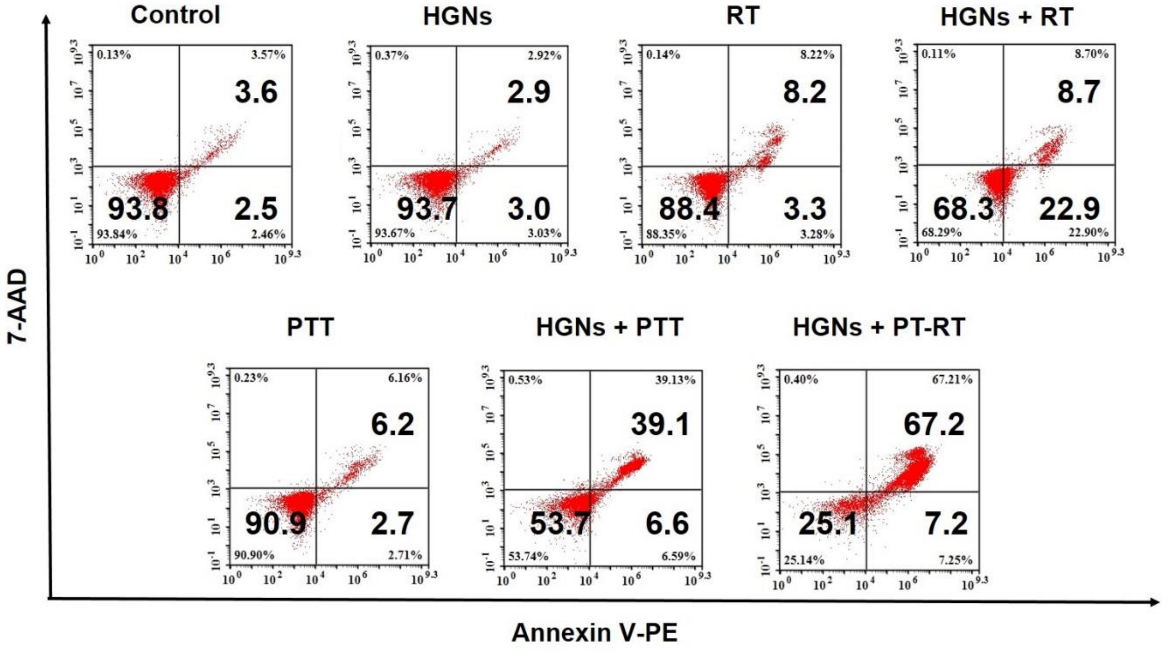

Figure S9. Flow cytometry-based apoptosis analysis of PANC-1 cells treated with HGNs (100 μg mL^{-1}) combined with laser irradiation (808 nm, 1 W cm^{-2}, 3 min) and X-ray radiation (6 Gy).

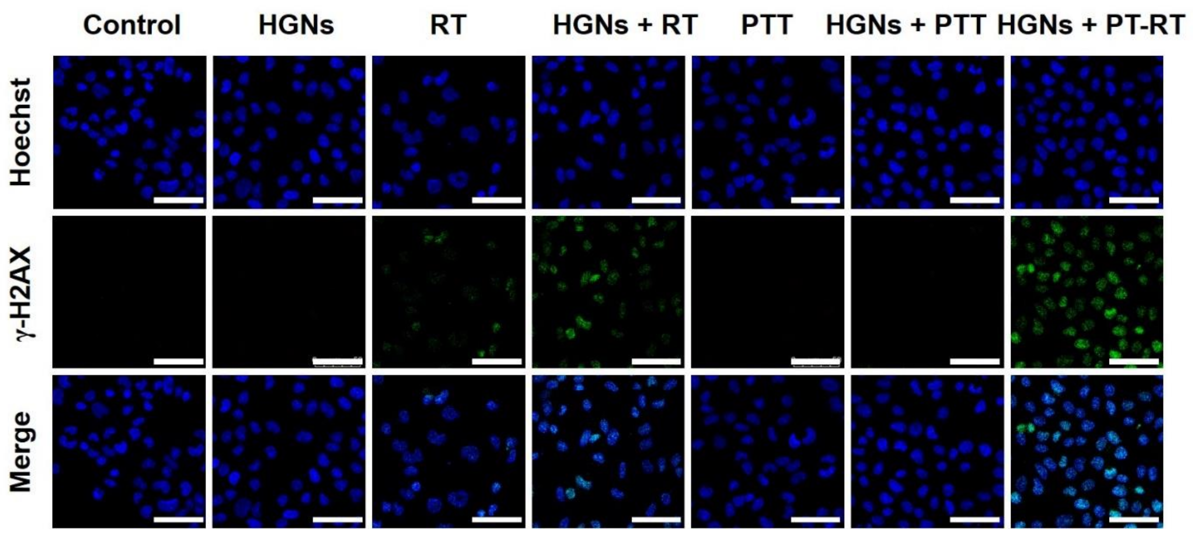

Figure S10. Fluorescence images of the nuclear condensation and DNA fragmentation in SW1990 cells induced by HGNs (100 μg mL^{-1}) with/without irradiation (808 nm, 1 W cm^{-2}, 3 min) with/without X-ray radiation (6 Gy), stained with Hoechst and γ-H2AX, respectively. Scale bars are 50 μm.

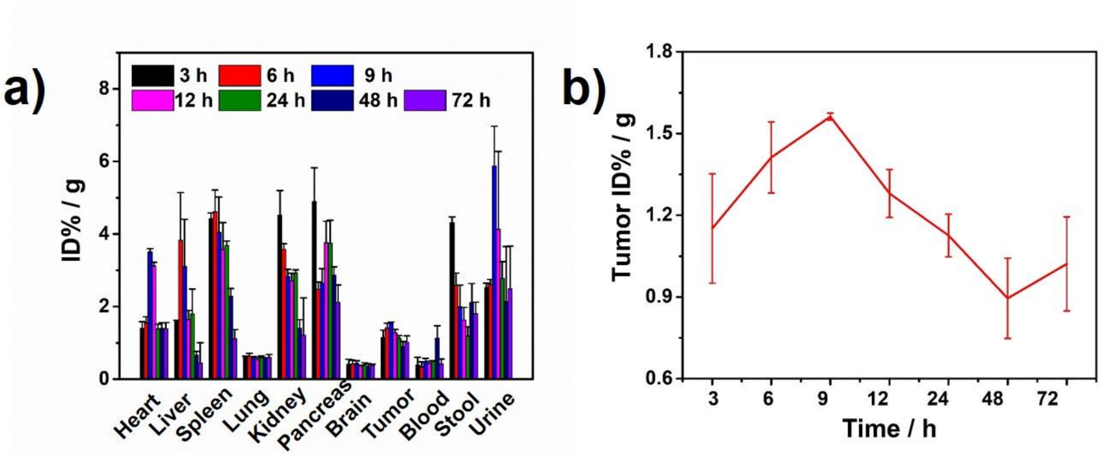

Figure S11. The biodistribution of HGNs in a) major organs, and b) tumor tissues was measured by ICP-MS.

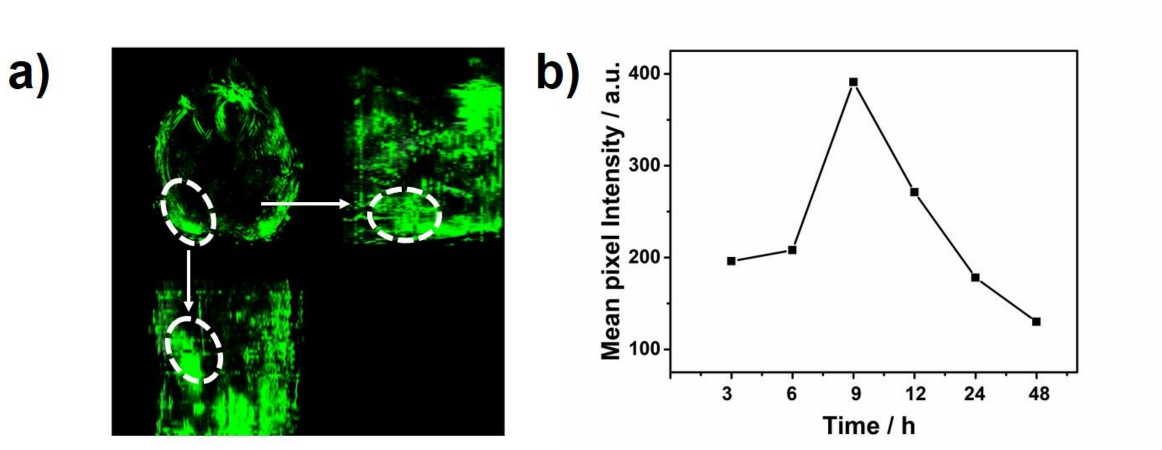

Figure S12. a) The PA image of tumor at 9 h. b) Corresponding PA signal at different time points.

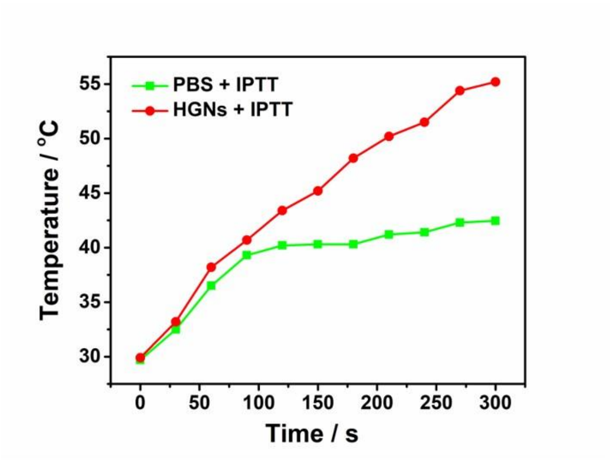

Figure S13. Temperature increase curves of tumor-bearing mice injected with HGNs (250 μg mL^{-1}, 100 μL) via the tail vein and then irradiated with a NIR laser (2.0 W cm^{-2}, 5 min), in comparison with PBS (100 μL) injection followed by NIR irradiation.

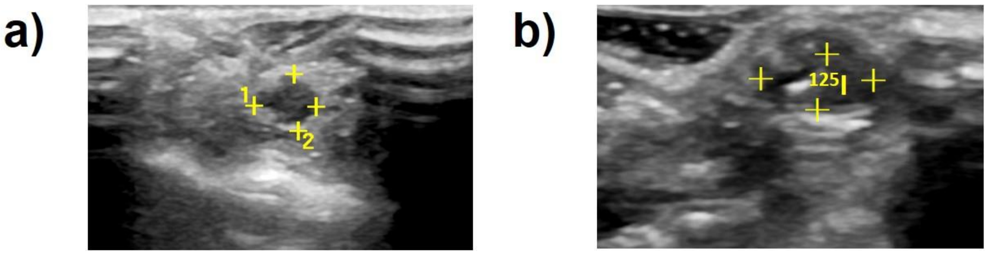

Figure S14. Ultrasound scan of SW1990 pancreatic tumor-bearing mice a) tumor before the procedure. b) One ^{125}I seed was implanted in the middle of the tumor and checked by ultrasound after the procedure.

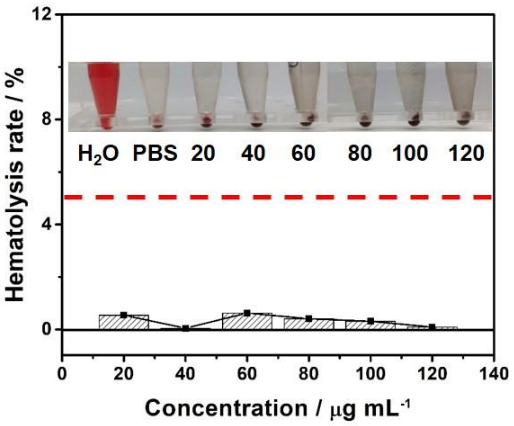

Figure S15. The hemolysis evaluation of HGNs at different concentrations (20, 40, 60, 80, 100, and 120 μg mL^{-1}). The inset is a photograph of red blood cells solution after incubated with HGNs.

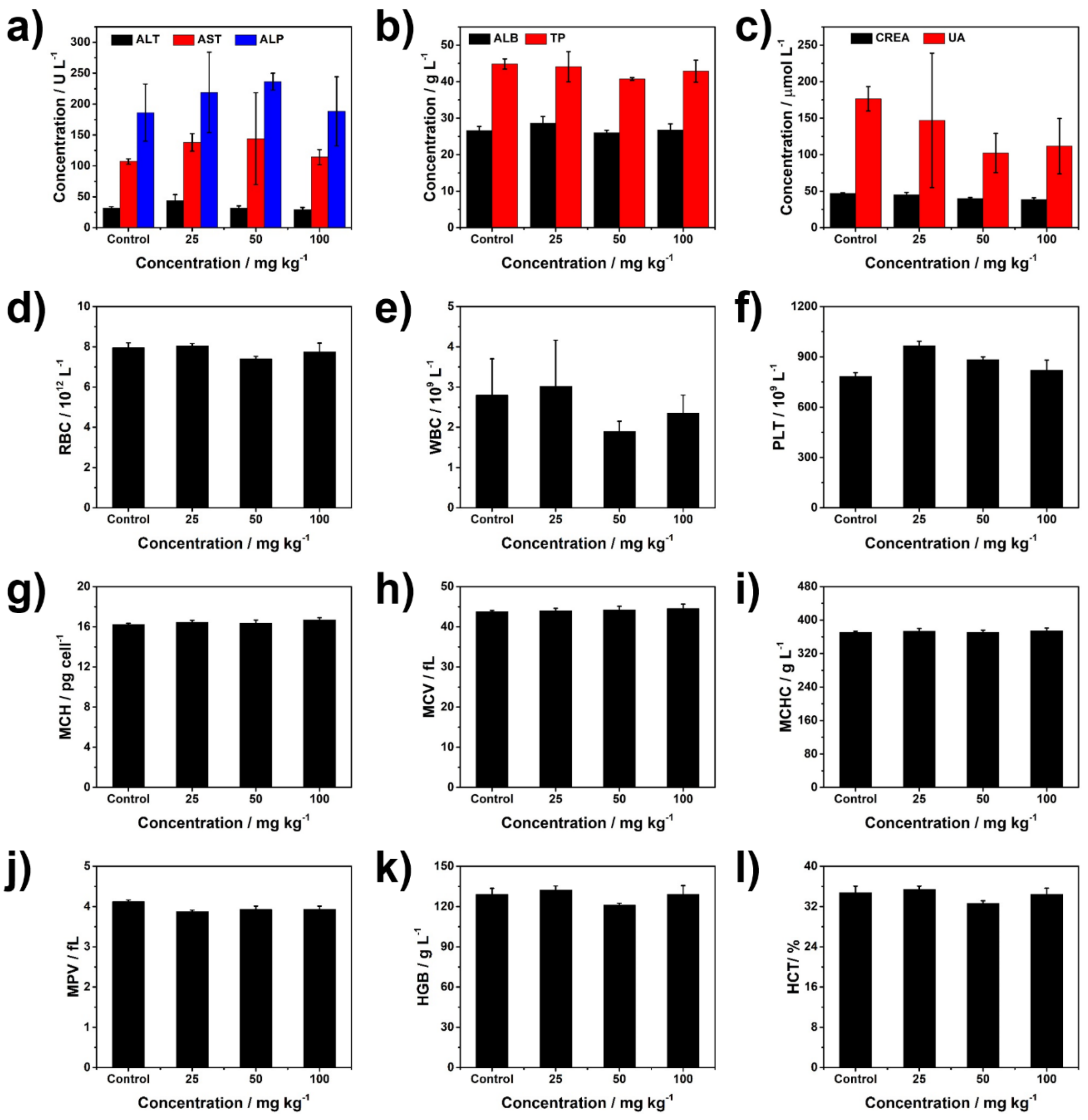

Figure S16. Blood biochemistry and hematology analysis of healthy BALB/c mice for 16 days after i.v. injection with HGNs at doses from 25 to 100 mg kg^{-1}. The main liver function markers: a) alanine aminotransferase (ALT), aspartate aminotransferase (AST), alkaline phosphatase (ALP), b) albumin (ALB), and total protein (TP), c) as well as kidney function makers containing creatinine (CREA) and uric acid (UA). The hematology analysis including d) red blood cells (RBC), e) white blood cells (WBC), f) platelets (PLT), g) mean corpuscular hemoglobin (MCH), h) mean corpuscular volume (MCV), i) mean corpuscular hemoglobin concentration (MCHC), j) mean platelet volume (MPV), k) hemoglobin (HGB), and l) hematocrit (HCT).

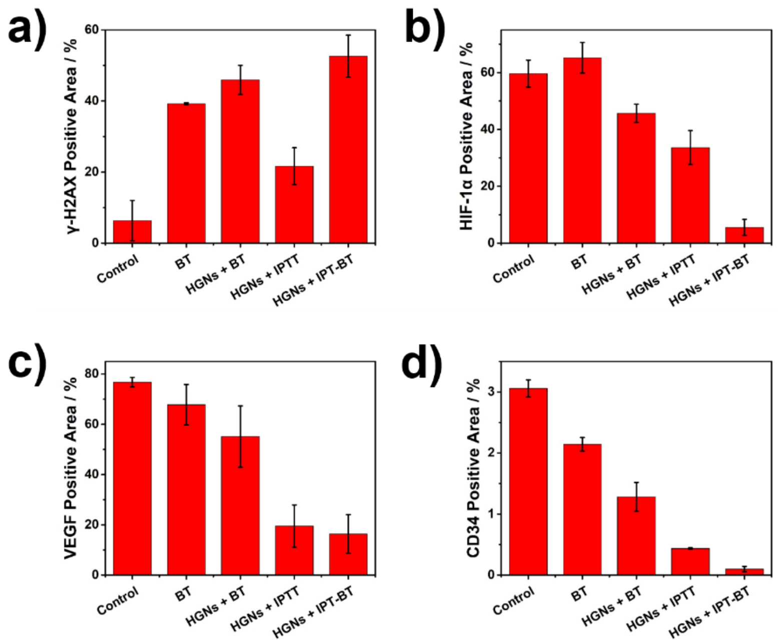

Figure S17. Quantitative measurements of immunohistochemical staining for different treatments.

Reference

[1] D. K. Roper, W. Ahn, M. Hoepfner, *J. Phys. Chem. C* **2007**, *111*, 3636.
